# Associations of short-term exposure to traffic-related air pollution with cardiovascular and respiratory hospital admissions in London, UK

**DOI:** 10.1136/oemed-2015-103136

**Published:** 2016-02-16

**Authors:** Evangelia Samoli, Richard W Atkinson, Antonis Analitis, Gary W Fuller, David C Green, Ian Mudway, H Ross Anderson, Frank J Kelly

**Affiliations:** 1 Department of Hygiene, Epidemiology and Medical Statistics, Medical School, National and Kapodistrian University of Athens, Athens, Greece; 2 St George's, University of London & MRC-PHE Centre for Environment and Health, London, UK; 3 King's College Analytical & Environmental Sciences Division, King's College London, London, UK

**Keywords:** Hospital Admissions, Short-term exposure, Time series analysis, Traffic-related pollution

## Abstract

**Objectives:**

There is evidence of adverse associations between short-term exposure to traffic-related pollution and health, but little is known about the relative contribution of the various sources and particulate constituents.

**Methods:**

For each day for 2011–2012 in London, UK over 100 air pollutant metrics were assembled using monitors, modelling and chemical analyses. We selected a priori metrics indicative of traffic sources: general traffic, petrol exhaust, diesel exhaust and non-exhaust (mineral dust, brake and tyre wear). Using Poisson regression models, controlling for time-varying confounders, we derived effect estimates for cardiovascular and respiratory hospital admissions at prespecified lags and evaluated the sensitivity of estimates to multipollutant modelling and effect modification by season.

**Results:**

For single day exposure, we found consistent associations between adult (15–64 years) cardiovascular and paediatric (0–14 years) respiratory admissions with elemental and black carbon (EC/BC), ranging from 0.56% to 1.65% increase per IQR change, and to a lesser degree with carbon monoxide (CO) and aluminium (Al). The average of past 7 days EC/BC exposure was associated with elderly (65+ years) cardiovascular admissions. Indicated associations were higher during the warm period of the year. Although effect estimates were sensitive to the adjustment for other pollutants they remained consistent in direction, indicating independence of associations from different sources, especially between diesel and petrol engines, as well as mineral dust.

**Conclusions:**

Our results suggest that exhaust related pollutants are associated with increased numbers of adult cardiovascular and paediatric respiratory hospitalisations. More extensive monitoring in urban centres is required to further elucidate the associations.

**What this paper adds**

- Little is known about the relative contribution of the sources and constituents to traffic-related exposure health effects.
- We selected metrics, from an extensive database, indicative of traffic sources.
- Exhaust-related metrics were associated with adult (15–64 years) cardiovascular and paediatric (0–14 years) respiratory hospitalisations.
- Aluminium, mineral dust tracer, was associated with adult cardiovascular admissions and respiratory hospitalisations mainly among those >15 years.
- Multipollutant models indicate independence of associations from different sources.

## Introduction

Epidemiological research has provided ample evidence for the adverse health effects of outdoor air pollution, mostly related to particulate pollution.^1^ Nevertheless, there remain significant gaps in our understanding of the most harmful constituents of ambient particles and their sources.^1^ ^2^ In urban areas, traffic-related pollution, comprising primary exhaust emissions from motor vehicles, road abrasion and tyre and brake wear, is of particular concern.^1^

While earlier epidemiological studies had identified associations between residence proximity to busy roads with outcomes such as cardiovascular and respiratory mortality,^3^ cardiovascular disease (CVD),^4^ lung function,^5^ the large scale European Study of Cohorts for Air Pollution Effects (ESCAPE) using near traffic exposure metrics such as particulate matter (PM) absorbance, nitrogen oxides (NOx) or traffic load and intensity failed to confirm associations with mortality,^6^ incidence of lung cancer,^7^ cerebrovascular^8^ or acute coronary events.^9^ Instead, this multicohort study provided evidence that traffic exposure metrics were associated with adverse paediatric respiratory outcomes,^10^ and elevated blood pressure or prevalent hypertension among adults.^11^ Epidemiological time series studies of short-term exposure and health effects have also reported mixed results for a range of health end points with individual pollutants, such as particles with aerodynamic diameter <2.5 μm (PM_2.5_), nitrogen dioxide (NO_2_), carbon monoxide (CO) or black carbon (BC).^11–17^ A previous study in London, UK^18^ suggested that certain particle components might be more important to specific diseases, pointing to particle number concentrations for CVD and secondary pollutants for respiratory outcomes. The biological mechanism for the traffic-related associations remains poorly understood, although some toxicological studies have suggested pulmonary and vascular inflammation as the relevant mechanism.^1^ ^2^

Previous investigation of the relative contribution of pollutants and sources using daily time-series analysis methods has been limited by dependence on data from routine monitors.^1^ The ClearfLo project^19^ characterised, in detail, the air pollution mixture in London between 2011 and 2012 and provided the opportunity to conduct daily time-series analyses focusing on specific sources, utilising data on the chemical composition of particles, estimation of the urban increment, as well as routine and study specific pollutant measurements. We selected a priori those metrics that best represented general traffic sources, diesel and petrol combustion, and non-exhaust sources (brake, tyre and road resuspension) for inclusion in a time-series analysis of respiratory and CVD daily emergency hospital admissions.

## Data and methods

### Data

Daily counts of emergency hospital admissions in London, UK between 2011 and 2012 were constructed from individual records of hospital admission obtained from the Hospital Episode Statistics (HES) system. Outpatient visits, elective admissions and visits to emergency department were not included. Based on the primary discharge diagnosis, daily numbers of admissions for CVD (International Classification of Diseases, 10th revision—ICD-10: I00-I99) for those aged 15–64 (adult) and 65+ years (elderly), and respiratory diseases (ICD-10: J00-J99) for those aged 0–14 years (paediatric), adult and the elderly were calculated.

Using data collected from the ClearfLo project,^19^ supplemented by local measurements made at the North Kensington urban background site, we assembled a database of metrics for 2011–2012, that included daily concentrations of particle mass (for particles with aerodynamic diameter <10 μm (PM_10_), or PM_2.5_), as well as particle composition (carbon, anions and metals) and gases (NO_2_, NOx, CO, sulfur dioxide (SO_2_) and ozone (O_3_)). All concentrations were based on 24 h averages except for CO and O_3_ for which the maximum 8 h moving average was computed. Daily concentrations of NOx, CO, BC and EC attributable to London sources rather than air mass transport were estimated by calculating the urban increment between North Kensington and two monitoring sites in the rural area around London dependent on wind direction each day.^20^

We then adopted a hypothesis-driven approach to the analyses. Based on a review of the literature on source identification, the London atmospheric emissions inventory^21^ and analysis of temporal trends and correlations we selected, a priori, pollutants to represent specific traffic sources (see online supplementary annexes 1–3 for detailed description and justification of selected pollutants, including correlation coefficients). In brief: (1) NOx was selected as a general traffic indicator, as 47% of it is emitted by road transport;^21^ (2) CO was selected as a proxy for emissions from petrol vehicles in London, as the contribution from petrol cars ranges from 0.07% to 0.9%, as compared with 0.01% to 0.07% for diesel vehicles;^22^ (3) elemental carbon (EC) in PM_10_ and black carbon (BC) in PM_2.5_ were chosen as indicators of diesel exhaust as studies of real-world vehicle emissions in London have demonstrated that diesel vehicles are overwhelmingly the largest emitters of EC and BC;^23^ (4) copper (Cu) in PM_10_ was selected as the indicator of brake-generated particles, as it is the most abundant element in brake linings and is found in high abundance in brake dust;^24^ (5) zinc (Zn) to reflect tyre-generated particles;^24^ and (6) aluminium (Al) as a marker of dust resuspension, as it occurs in sufficient quantities and is not identified in other sources.^25^ Regulated pollutants (PM_10_, PM_2.5_, NO_2_, SO_2_ and O_3_) were also selected for comparability with previous findings and mutual control in multipollutants’ models. There were few missing values in the pollution time-series (ranging from 0% for particle mass concentrations and CO to 19% for EC urban increment).

Time series of daily temperature (°C, mean) and relative humidity (%) were obtained from a meteorological tower located close to the North Kensington monitoring site.

## Methods

We investigated the associations between short-term exposure to traffic-related pollutants and daily hospital admissions using Poisson regression models allowing for overdispersion. The model was of the form:
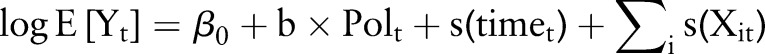
where E[Y_t_] is the expected value of the Poisson distributed variable Y_t_ indicating the daily outcome count on day t with Var(Y_t_)=φE[Y_t_], φ being the overdispersion parameter, time_t_ the continuous variable indicating the time (day) of event (from 1 to 731), Pol_t_ the pollutant concentration on day t, X_it_ the value of confounder X_i_ on day t, and s denotes smoothing functions. We used penalised regression splines^26^ as smoothing functions *s* to capture the association between time-varying covariates, calendar time and health outcome. Degrees of freedom (df) for long-term trends were based on the minimisation of the absolute value of the sum of the partial autocorrelations function (PACF) of the residuals from lags 1 to 30, imposing a minimum of 3 df per year. We also included dummy variables for the day of the week and public holidays. For the analysis of respiratory admissions among ages 0–14 and 15–64 years we included an extra dummy variable denoting the month of August, as the decrease in the respiratory admissions at this period could not be sufficiently captured by the smooth term of seasonality. We controlled for mean daily temperature and relative humidity to address any potential confounding effects of weather. For temperature control we applied a natural spline with 3 df for same day's exposure (lag 0) to capture the effect of high temperatures on health, while to capture the health effects of lower temperatures we used the corresponding function on the average of the six previous days exposure (lags 1–6), as these terms minimised the Akaike's Information Criterion. For relative humidity adjustment, we included a linear term for the average of the same and the two previous days, sufficient to capture any residual weather confounding. When we investigated the associations with EC/BC and metal components of particles, we also controlled for particle mass (PM_10_ for EC and metals and PM_2.5_ for BC), as a way to distinguish the effect of the particular constituent from the rest.^27^

We decided *a priori* which lags of the pollutants to be included in the models: previous day's exposure for CVD admissions (lag1) and previous 2 days’ exposure for respiratory admissions (lag2), based on prior indications of longer lags for respiratory outcomes.^18^ To investigate any prolonged effects, we additionally applied unconstrained distributed lag models for the previous week's exposure (lags 0–6).

We applied multipollutant models after considering the correlations between pollutant pairs (see online supplementary annex 3). We included pollutants in a model in cases when the correlation was below 0.7. Specifically we applied two pollutant models to test the robustness of the associations with gases and three pollutant models for EC/BC and metal components of particles. For gases, the second pollutant entered in the model was selected in order to test the hypothesis of independent effects between traffic or long-range transport-related metrics (NOx or CO controlling for PM_2.5_ or EC, SO_2_ and O_3_). For EC/BC and metals, for which already the corresponding particle mass was controlled in the model, we additionally adjusted for NOx and CO. In order to minimise the correlation between the three metrics, instead of adding the third metric in the model, we initially regressed the gaseous pollutant on particle mass (PM_10_ or PM_2.5_) and consequently entered the model residuals in the model to adjust for any remaining effect not attributed to particles.^27^

We investigated the associations by season defined as warm (April–September) and cool (October–March) period to test the hypothesis of effect modification due to differential sources and exposure misclassification between periods. For these analyses we controlled for seasonality and long-term trends using indicator variables per month per year of the study, while the rest of the confounding control was the same as in the annual model.

All models were fit in R V.3.0.3 (R development Core Team (2011), ISBN 3-900051-07-0, URL http://www.R-project.org) using the package *mgcv (V.1.7–28)*. Results in tables and plots are presented as per cent change associated with an IQR increase in the pollutant's concentration.

## Results

Table 1 presents descriptive statistics for the daily number of hospital admissions, daily concentrations for the pollutants and meteorological parameters. The greater London area had a population of 9 787 426 inhabitants (2011 Census). The mean number of hospital admissions per day varied from 104 for CVD in the elderly to 46 for paediatric respiratory diseases. Mean PM concentrations were 18.4 μg/m^3^ for PM_10_ and 12.2 μg/m^3^ for PM_2.5_, while mean concentrations of gaseous pollutants were 55.3 μg/m^3^ for NOx, 1.8 μg/m^3^ for SO_2_ and 0.3 mg/m^3^ for CO. The urban increment of NOx, CO, EC and BC accounted for most of the measured concentration, showing them to be dominated by urban sources, with the exception of CO (mean concentration 0.3 mg/m^3^, with estimated urban increment of 0.1 mg/m^3^). Higher concentrations of traffic-related pollutants were recorded during the cool period (see online supplementary annex 3), however the roadside enrichment factors were lower, when compared with the warm period. Cool period enrichment factors for NOx, BC, EC and CO were 3.5, 4.5, 4.2 and 1.1 respectively, increasing to 6.8, 7.2, 7.3 and 1.8 during the warm months, implying that roadside sources were more dominant in the warm period, even though total pollutant concentrations were lower. Overall period correlations among pollutants ranged from 0.2 (correlations with O_3_) to >0.9 (see online supplementary annex 3). Specifically, correlations of CO were: 0.83 with NOx, 0.77 with BC and 0.62 with Cu. Correlations using the urban increment of the pollutants were substantially smaller than those with the total measured concentration; for example, the correlation between NOx and CO was 0.83, but was reduced to 0.41 when only the urban increment was considered.

**Table 1:** Descriptive characteristics of hospital admissions counts, traffic-related pollutants and meteorological variables in London, UK for 2011–2012

	Number of days	Mean	Median	IQR (75th–25th centile)	90th Centile
*Hospital admissions*					
Cardiovascular (years)					
15–64	731	56	57	25	71
65+	731	102	104	37	124
Respiratory (years)					
0–14	731	46	45	23	72
15–64	731	64	63	16	81
65+	731	96	91	28	125
*Pollutants (μg/m^3^; CO in mg/m^3^)*					
General traffic indicator					
NOx	706	55.3	41.2	41.3	106.5
NOx urban increment	703	42.5	30.8	33.1	84.4
Petrol vehicle exhaust					
CO	729	0.3	0.3	0.2	0.5
CO urban increment	724	0.10	0.08	0.09	0.21
Diesel vehicle exhaust					
EC (in PM_10_)	682	1.0	0.8	0.8	1.9
EC urban (in PM_10_)	590	0.8	0.6	0.5	1.4
BC (in PM_2.5_)	702	1.5	1.2	1	2.8
BC urban (in PM_2.5_)	629	0.9	0.7	0.6	1.8
Vehicle non-exhaust					
Cu (in PM_10_)	677	0.0093	0.0072	0.0075	0.0176
Zn (in PM_10_)	677	0.012	0.0087	0.0091	0.0246
Al (in PM_10_)	677	0.076	0.0555	0.0605	0.1528
Regulated pollutants (μg/m^3^)					
PM_10_	729	18.4	15.0	10	32.5
PM_2.5_	730	12.2	9.0	8	25.0
NO_2_	706	36.3	33.3	23.7	58.1
SO_2_	717	1.8	1.8	2.2	3.6
O_3_	716	55.4	54.7	30.3	85.9
Meteorological parameters					
Mean temperature (°C)	731	11.70	11.70	7.5	18.10
Relative humidity (%)	731	76.43	78.00	14.6	88.50

PM, particulate matter; PM_2.5_, particles with aerodynamic diameter <2.5 μm; PM_10_, particles with aerodynamic diameter <10 μm.

Table 2 presents the per cent change in hospital admissions for an IQR increase in the concentrations of the traffic-related pollutants following single day exposure (lag1 for CVD and lag2 for respiratory diagnoses). Associations with regulated pollutants are presented in online supplementary annex 4 as these were not the focus of the present analysis. Table 3 presents the per cent change following weekly exposure (lags 0–6).

**Table 2:** Per cent change (and 95% CIs) in cardiovascular and respiratory hospital admissions associated with an IQR increase in traffic-related pollutants after acute exposure (lag 1 for cardiovascular and lag 2 for respiratory diagnoses) in London, UK for 2011–2012

	CVD admissions % (95% CI)		Respiratory admissions % (95% CI)		
Indicator/pollutants	15–64 years	65+ years	0–14 years	15–64 years	65+ years
General traffic					
NOx	0.86 (−0.28 to 2.02)	−0.32 (−1.19 to 0.56)	1.06 (−0.43 to 2.57)	−0.81 (−1.92 to 0.31)	−1.76 (−2.77 to −0.74)
NOx—urban	0.92 (−0.15 to 2.00)	−0.15 (−0.97 to 0.67)	1.07 (−0.31 to 2.46)	−0.60 (−1.64 to 0.46)	−1.51 (−2.45 to −0.55)
Petrol vehicle exhaust					
CO	1.59 (0.12 to 3.07)	−0.60 (−1.71 to 0.52)	1.05 (−0.96 to 3.10)	−1.11 (−2.57 to 0.36)	−2.10 (−3.43 to −0.75)
CO—urban	0.95 (−0.06 to 1.98)	−0.16 (−0.93 to 0.62)	0.97 (−0.40 to 2.36)	0.21 (−0.80 to 1.24)	−0.59 (−1.52 to 0.34)
Diesel vehicle exhaust					
EC	1.63 (0.15 to 3.13)	0.18 (−0.94 to 1.32)	0.72 (−1.22 to 2.70)	−0.19 (−1.63 to 1.27)	−0.88 (−2.19 to 0.45)
EC—urban	1.28 (0.17 to 2.40)	0.14 (−0.72 to 1.00)	1.27 (−0.21 to 2.78)	−0.03 (−1.12 to 1.08)	−0.05 (−1.04 to 0.96)
BC	1.65 (0.11 to 3.21)	0.56 (−0.61 to 1.74)	0.86 (−1.13 to 2.88)	−0.20 (−1.71 to 1.33)	−1.09 (−2.47 to 0.31)
BC—urban	0.74 (−0.47 to 1.97)	−0.04 (−0.97 to 0.89)	1.08 (−0.50 to 2.68)	0.44 (−0.78 to 1.66)	−0.01 (−1.10 to 1.09)
Vehicle non-exhaust					
Cu	1.39 (−0.03 to 2.83)	0.06 (−1.02 to 1.16)	0.08 (−1.81 to 2.01)	−1.18 (−2.60 to 0.26)	−1.60 (−2.89 to −0.28)
Zn	0.08 (−1.25 to 1.42)	0.16 (−0.85 to 1.18)	−0.92 (−2.72 to 1.47)	−0.38 (−1.73 to 1.00)	−0.73 (−1.96 to 0.52)
Al	0.43 (−1.18 to 2.07)	−1.14 (−2.35 to 0.09)	0.19 (−2.22 to 2.66)	0.82 (−0.84 to 2.50)	1.38 (−0.15 to 2.94)

EC/BC and metals are adjusted for PM mass.

CVD, cardiovascular disease; PM, particulate matter.

**Table 3:** Per cent change (and 95% CIs) in cardiovascular and respiratory hospital admissions associated with an IQR increase in traffic-related pollutants after weekly exposure (lags 0–6) in London, UK for 2011–2012

Indicator/pollutants	CVD admissions % (95% CI)		Respiratory admissions % (95% CI)		
	15–64 years	65+ years	0–14 years	15–64 years	65+ years
General traffic					
NOx	−0.92 (−2.98 to 1.18)	0.20 (−1.38 to 1.80)	4.01 (0.76 to 7.37)	−1.67 (−3.70 to 0.39)	−5.13 (−6.98 to −3.24)
NOx—urban	−0.37 (−2.43 to 1.73)	0.45 (−1.12 to 2.04)	3.86 (0.67 to 7.16)	−0.95 (−2.97 to 1.11)	−4.77 (−6.61 to −2.90)
Petrol vehicle exhaust					
CO	1.03 (−1.85 to 3.99)	−1.18 (−3.31 to 1.01)	4.94 (0.11 to 10.00)	−2.51 (−5.29 to 0.36)	−8.81 (−11.28 to −6.28)
CO—urban	2.52 (0.17 to 4.92)	−1.08 (−2.82 to 0.69)	4.02 (0.24 to 7.93)	−0.31 (−2.64 to 2.07)	−3.68 (−5.87 to −1.43)
Diesel vehicle exhaust					
EC	1.39 (−1.59 to 4.45)	2.36 (0.05 to 4.73)	1.64 (−2.85 to 6.35)	−1.62 (−4.54 to 1.39)	−2.97 (−5.69 to −0.18)
EC—urban	1.46 (−0.93 to 3.91)	1.65 (−0.18 to 3.52)	2.59 (−1.45 to 6.79)	0.56 (−1.96 to 3.14)	−0.02 (−2.38 to 2.40)
BC	0.13 (−2.84 to 3.19)	1.49 (−0.82 to 3.86)	4.01 (−0.70 to 8.94)	−0.87 (−3.84 to 2.21)	−3.11 (−5.91 to −0.23)
BC—urban	0.49 (−3.00 to 4.11)	2.13 (−0.57 to 4.91)	1.34 (−3.76 to 6.70)	0.64 (−2.88 to 4.29)	−0.40 (−3.61 to 2.91)
Vehicle non-exhaust					
Cu	2.23 (−0.39 to 4.91)	1.11 (−0.88 to 3.14)	−5.60 (−9.28 to −1.77)	−1.98 (−4.58 to 0.70)	−2.13 (−4.57 to 0.36)
Zn	0.91 (−1.96 to 3.87)	0.12 (−2.08 to 2.37)	−4.26 (−8.24 to −0.11)	−3.17 (−6.00 to −0.25)	−2.53 (−5.15 to 0.16)
Al	0.75 (−2.01 to 3.59)	0.32 (−1.78 to 2.47)	−6.43 (−11.08 to −1.55)	2.46 (−0.50 to 5.50)	4.49 (1.62 to 7.473)

EC/BC and metals are adjusted for PM mass.

CVD, cardiovascular disease; PM, particulate matter.

### General traffic indicator

Both the total measured concentration at North Kensington and the urban increment of NOx displayed positive associations (table 2) with CVD adult admissions and paediatric respiratory admissions. Moreover, when considering longer lags of exposure (lags 0–6, table 3) NOx presented an adverse association with CVD admissions in the elderly, 0.20% increase (95% CI −1.38% to 1.80%) for an IQR increase in total measured NOx and 0.45% increase (95% CI −1.12% to 2.04%) for its urban increment. Associations between measured concentrations of NOx, and its urban increment, and paediatric respiratory hospital admissions were also observed, 4.01% (95% CI 0.76% to 7.37%) and 3.86% (95% CI 0.67% to 7.16%) respectively. Negative associations, for both the total and the urban part concentration, irrespective of the lag structure, were observed for respiratory admissions in the elderly population.

### Petrol vehicle exhaust indicator

CO, total concentration and urban increment, was associated with CVD adult admissions, more strongly following lag 1 exposure for CO (1.59% increase (95% CI 0.12% to 3.07%), table 2) and lags 0–6 exposure for its urban increment (2.52% increase (95% CI 0.17% to 4.92%), table 3). Positive associations were also estimated for paediatric respiratory admissions that became higher for longer exposures, while the opposite patterns were observed for respiratory admissions in the elderly.

### Diesel vehicle exhaust indicators

Adverse associations with all CVD outcomes were estimated for EC following acute exposure (table 2) among adults for the total measured mass (1.63% increase, 95% CI 0.15% to 3.13%) and for the urban increment (1.28%, 95% CI 0.17% to 2.40%). Similar adverse associations were also observed after prolonged exposure (lags 0–6, table 3) among the elderly for total measured mass (2.36% increase, 95% CI 0.05% to 4.73%) and the urban increment (1.65%, 95% CI −0.18% to 3.52%). Positive associations were also estimated for paediatric respiratory hospital admissions (tables 2 and 3); while adverse associations among the adult and elderly age groups were estimated only for weekly exposures to the urban increment (table 3).

BC, both total and urban increment, also displayed positive associations with all CVD outcomes (tables 2 and 3), except with CVD admissions in the elderly group for lag 1, for which no association was observed. Consistent positive associations were also noted for all respiratory outcomes following either acute (table 2) or weekly (table 3) exposure to the urban BC contribution, while exposure to the total measured BC was associated with paediatric respiratory admissions.

### Vehicle non-exhaust (mineral dust, brake and tyre wear) indicators

We found positive associations between Cu, Zn and Al in PM_10_ and CVD hospital admissions in adults (tables 2 and 3). Al was positively associated with respiratory hospital admissions across all age groups and exposure periods studied (tables 2 and 3) with the single exception for lags 0–6 exposure and paediatric respiratory admissions. Conversely, we observed negative associations between Cu and respiratory hospitalisations irrespective of age and lag.

In summary, all traffic-related pollutants displayed adverse associations with CVD admissions in adults after a single day exposure, while positive effect estimates for EC and BC were observed for CVD admissions among the elderly. Considering weekly exposures, effect estimates for CVD admissions were generally lower for adults compared with the elderly. We observed positive associations with all pollutants except Zn on paediatric respiratory admissions. The highest effect estimates were for the association with the EC (1.27% increase) and BC (1.08%) urban increment concentrations. Longer exposure resulted in higher estimates for paediatric respiratory admissions (except for metals). Only the urban increments of CO and BC, and Al displayed an association with respiratory admissions among adults, with only Al showing an association in the elderly (1.38% increase).

In general, effect estimates of traffic pollutants on hospital admissions displayed a consistent decreasing trend with increasing age, while associations were higher with CVD as compared with respiratory emergency admissions. For example an IQR increase in BC was associated 1.65% increase in CVD admissions in adults compared with a 0.56% increase in the corresponding age group for respiratory admissions.

Effect estimates for regulated pollutants (PM_10_, PM_2.5_, NO_2_ and SO_2_) also supported findings of effects only within the younger age group, namely for adult CVD and paediatric respiratory admissions (see online supplementary annex 4), while only exposure to O_3_ was related to effects among the older age groups (CVD 65+ years and respiratory >15 years).

The effect estimates derived from single pollutant models were robust to adjustment of other pollutants (see online supplementary annex 5). EC and Al presented the most consistent associations, while there was indication of confounding between CO and EC/BC that lowered the estimates; these nevertheless remained positive.

**Figure 1:**
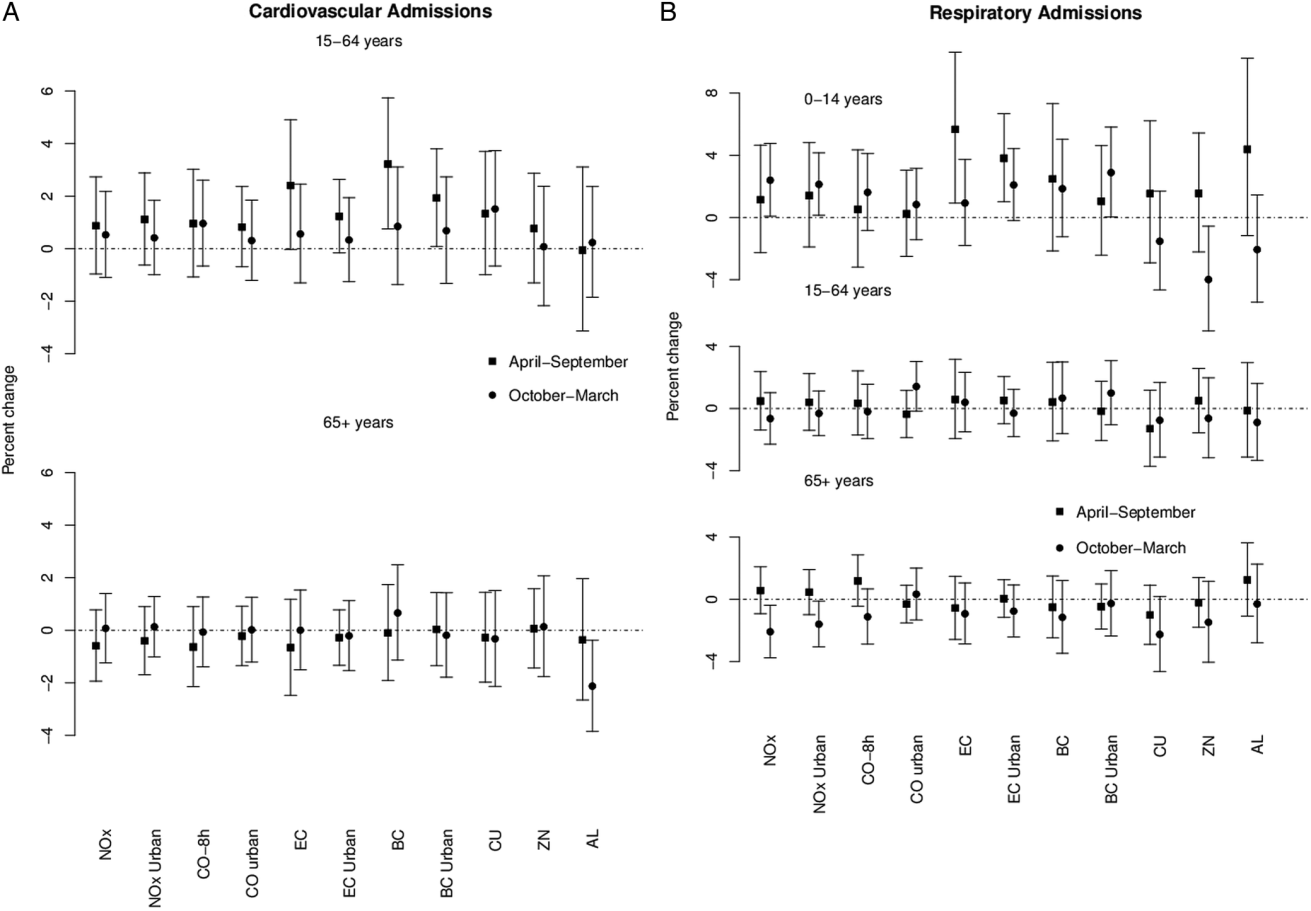
Per cent change (and 95% CIs) in cardiovascular (A) and respiratory (B) hospital admissions associated with an IQR increase in traffic pollutants after single day exposure (lag 1 for cardiovascular and lag 2 for respiratory diagnoses) in London, UK for 2011–2012 during the warm (April–September) and cool (October–March) period of the year. EC/BC and metals are adjusted for particle mass.

Figure 1 presents the per cent change in hospital admissions by warm and cool period of the year for an IQR following single day exposure in traffic-related pollutants. Effect estimates (and 95% CIs) are presented in the online supplementary annex 6. The seasonal analysis revealed higher effect estimates during the warmer period of the year, except for CVD admissions in the elderly, although in general there was no difference in the estimates between periods. The associations of EC with CVD admissions for adult and paediatric respiratory admissions were higher during the warm period, as was also the case for Al on the latter outcome.

## Discussion

We investigated associations between short-term exposure to an a priori selection of traffic-related pollutants and CVD and respiratory hospital admissions in London, UK. We found consistent positive associations between EC, BC and Al in PM_10_ after a single day exposure and CVD outcomes in adults and with paediatric respiratory admissions. Furthermore, 7 days averages for these pollutants were associated with CVD admissions in the elderly. These particular associations were larger in the warm period of the year compared with the cooler period although the differences did not achieve statistical significance.

The main strengths of our study are the range and the quality of the pollution metrics assembled from routine and augmented monitoring at a central urban background site, plus the capacity to address the urban increment for metrics where parallel monitoring was performed at rural locations. Assembling a database with such an extensive range of constituents is a major, and possibly unique, aspect of our study—an example of translational research linking laboratory with epidemiology. Moreover, London, due to its large size, provides the adequate number of mean daily counts necessary for the variability of the time series. We adopted a hypothesis-driven approach to select a limited number of the available metrics to reflect potential traffic sources to the urban air shed. This selection was informed by a detailed review of the literature, data completeness and the observed correlation structure between the available metrics. This *a priori* selection of traffic indicators, as well as predefined lags for CVD and respiratory admissions limited the number of uninformed comparisons performed and we believe strengthens the robustness of our findings.

Although a limitation of the present study is the relatively small sample size (2 years) for a time-series design, this was compensated for by the completeness of the pollutant measurements over the study period and the large baseline population. An inherent drawback of time-series studies is the misclassification induced due to the use of fixed monitors to estimate the population's exposure; in this case a single fixed site in North Kensington, in inner London. Nevertheless, previous analyses have identified positive associations using this urban background site.^23^ We tested the sensitivity of the effect estimates of the regulated pollutants (PM_10_, PM_2.5_, NO_2_, CO and O_3_) obtained from the North Kensington site to the ones from the average of the daily measurements from available fixed monitoring stations scattered around London and the results were comparable, for example, an IQR increase in NO_2_ from North Kensington was associated with 1.00% (95% CI −0.87% to 2.91%) change in CVD admissions 15–64 years, while the average of all urban and suburban background monitors was associated with a 0.77% (95% CI (−1.01% to 2.58%)) change. Nevertheless, due to possible differences in the spatial variability of the traffic-derived pollutants measured in North Kensington, there remains a chance of residual confounding, though these are not likely to rule out causation, especially in pollutants that display consistent associations.^23^ Although multiple comparisons for detection of associations may have resulted in inflation of type I error, we chose not to correct for this but instead identified associations with pollutants that were consistent across different outcomes. Finally, the chosen metrics did not entirely represent traffic sources as they partly originate from other sources. The degrees of specificity for traffic sources can be gauged from the roadside enrichment factors (see online supplementary annex 1) which varied from 1.3 for Al and Zn and 1.4 for CO, indicating a relatively low contribution from traffic, to 4.6 for NOx, 4.7 for Cu and 5.6 for BC, where traffic sources made a greater contribution. The contribution from traffic also varies by season; for instance it was lower for NOx in winter when space heating also contributed. Differential pollutant dispersion between seasons also has an effect in addition to source changes.

The urban increments of EC and BC provided lower effect estimates for CVD admissions and higher estimates for respiratory admissions compared with the corresponding total concentrations. There were indications of associations with both pollutants among people below 65 years, while more prolonged exposure was associated with CVD outcomes among the elderly (65+ years). In general, the BC urban increment revealed a more consistent pattern. BC and EC were selected a priori as diesel exhaust markers (see online supplementary annex 1) and the results from the multipollutants’ analysis indicated that although there was some confounding when adjusting for CO, as a petrol exhaust marker, the adverse associations generally remained, pointing towards independent effects of different sources. Recent reviews^21^ ^28^ on BC health effects concluded that, although there is sufficient evidence on short-term exposure and effects on cardiopulmonary admissions, the toxicological evidence suggested that BC may not be a the major directly toxic component of fine PM. BC may operate as a universal carrier of a wide variety of chemicals of varying toxicity to the lungs, which may then induce adverse effects within and beyond the respiratory system, the body's major defence cells and possibly the systemic blood circulation. Alternatively, BC may act as a surrogate of true causal pollutants correlated through a common source. Recent experimental studies have demonstrated systemic effects of BC on arterial blood pressure responses^29^ and of diesel exhaust itself on haemoconcentration and thrombocytosis—potentially important determinants of acute CVD events.^30^

CO, selected as a petrol indicator, was associated with an increased risk of CVD admissions among adults. The urban increment in CO also provided consistent associations with adult respiratory admissions. There was some indication of confounding with EC and BC (as diesel markers) but the association was still apparent. A meta-analysis of single or multicities results^31^ reported evidence for adverse effects of short-term CO exposures on hospitalisations due to respiratory or diagnoses-specific CVD admissions, and Bell *et al*^32^ also found evidence of an association with risk of CVD hospitalisations in 126 US counties. Notably, although in both studies concentrations of CO were well below the EU air quality standards (http://ec.europa.eu/environment/air/quality/standards.htm) the associated health effect estimates were high compared with the other pollutants.

We found little evidence of effects of NOx as a general traffic marker, although there were positive associations with CVD adult admissions that persisted in two pollutant models. The increase in CVD is compatible with the higher systolic blood pressure reported by Kubesch *et al*^29^ following short-term exposure to traffic-related air pollution. We also observed positive but not statistically significant associations with paediatric respiratory admissions (1.06% increase per IQR). Previous panel studies on children with asthma have reported adverse effects of exposure to NOx,^1^ while Iskandar *et al*^33^ using a time-series design also reported increases in hospital admissions for asthma among 0–18 years. On the other hand, consistent protective associations were found with respiratory admissions among the elderly. Protective associations are not supported by plausible biological mechanisms, but it is possible that elderly patients with respiratory conditions may avoid outdoor exposure as a result of public health warning messages (air pollution forecasts are incorporated into weather forecasts in the UK) or use prophylactic medications and hence modify the associations observed in our study. Most previous times-series studies have focused on NO_2_. However, NOx may also reflect NO_2_ effects due to their high correlation (r=0.90), further supported by their similar effect estimates. The WHO review^1^ concluded that there is consistent epidemiological evidence and some mechanistic support for causality of some NO_2_ direct effects. In our analysis adjustment for particles, sulfates or gaseous regulated pollutants increased our effect estimates supporting the plausibility of the reported associations.

Cu, Zn and Al in PM_10_ were selected as markers of non-exhaust traffic contributions to PM_10_. Al, selected as a mineral dust tracer, demonstrated adverse associations with CVD admissions in adults (0.43% increase per IQR) and with respiratory admissions in adults (0.82% per IQR increment) and elderly (1.38% per IQR increment). Bell *et al*^34^ also reported effects of Al on respiratory admission among the elderly, which is the association with the highest effect estimate also in our analysis. There was also some evidence of an association between Cu as tracer of brake generated particles and CVD admissions. Basagaña *et al*^35^ using data from five Southern European cities reported adverse associations of Cu on CVD morbidity, that were higher than the ones found in London (1.94% increase per IQR), but did not persist after adjustment for PM mass as in our data. There is toxicological evidence for the biological mechanism of effects, as Cu and Zn have both been linked to a decrease in spontaneous beat rate, vasoconstriction and vasodilatation.^36^ However, we did not find convincing evidence for an association between Zn and CVD or respiratory admissions, although this metal has been studied and associations have been previously reported.^1^ ^35^

The higher effect estimates of all traffic-related pollutants observed in the younger age groups, between 15 and 64 years for CVD and mainly in children 0–14 years for respiratory admissions, are of particular interest. The underlying mechanisms for the observed patterns may be attributed to age-specific different diagnoses, but also to moderation of pre-existing disease (especially CVD) in the elderly. This hypothesis is also supported by the indication of associations with CVD outcomes following longer periods of exposure. Few epidemiological studies on the effects of short-term exposure to air pollution have reported age modification patterns in hospitalisations. Using European data from the 1990s, Le Tertre *et al*^37^ found larger effects of particles on CVD admissions among the elderly, but since 2000 the increased use of statins and other medications for CVD diseases could potentially have modified this risk. Higher respiratory effects of particles in younger ages have been previously reported,^38^ though previous reports of particle-related effects on the elderly do not chimes with our current findings.^38^ ^39^ Finally, it is also possible that as a response to increased public awareness during the past decades on the health effects of air pollution and inclusion of air pollution levels and forecasts into the daily weather forecasts, sensitive subgroups such as the elderly with pre-existing conditions may have modified their time-activity patterns resulting in modification of effects.

We found higher effect estimates between the selected traffic-related pollutants (except for CO) and adult (15–64 years) CVD admissions and respiratory admissions among those below 65 years of age during the warmer period of the year compared with the cooler period. European epidemiological studies on the health effects of short-term exposure to air pollution have reported higher associations during the warmer period of the year,^14^ ^39^ often attributed to better exposure characterisation of the population. Nevertheless US studies^40^ have reported higher numbers of hospitalisations for the elderly during the cool period of the year suggesting that seasonal patterns may differ across age groups that potentially follow different activity patterns. Moreover, toxicity of particles originating from different sources may vary between seasons and locations. In the current analysis the roadside enrichment factors suggest that traffic sources are more dominant in summer months due to seasonal variation in sources and dispersion.

The results indicate consistent associations predominately with EC/BC but also to a lesser degree with CO and PM_10_ Al content, with CVD and respiratory admissions among younger age groups. Although the specific effect estimates are variable to the adjustment of other pollutants they remain largely consistent in direction, indicating the independence of effects from different traffic sources especially from diesel and petrol engines, as well as resuspended mineral dust. Supporting this argument, the cumulative effect of traffic-related pollution is not appropriately captured from a general indicator such as NOx, possibly due to differential patterns of pollutant correlations or associated effects. This conclusion is crucial for planning public health policies aiming at the reduction of air pollution effects.

Our findings point towards short-term exposure to exhaust rather than non-exhaust-related pollutants as the ones mostly associated with adverse effects on morbidity, previously attributed to traffic-related pollutants. As diesel-powered engines are the main urban source of EC and BC, which presented the most consistent indications, actions to further abate diesel emissions should be prioritised as part of policy measures for protection of public health. However, our results in respect to CO also suggest that there should also be stricter control of emissions from petrol combustion. The role of non-exhaust sources remains a concern however and more extensive monitoring of traffic pollution in urban centres is required to further elucidate the associations.

## Supplementary Material

Web supplement
