# Supplementary material for: Associations of short-term exposure to traffic-related air pollution with cardiovascular and respiratory hospital admissions in London, UK
Source: Occup Environ Med. 2016 Feb 16;73(5):300–7. doi: 10.1136/oemed-2015-103136 (PMC4853598; doi:10.1136/oemed-2015-103136)
Supplement: Web supplement [file oemed-2015-103136-s1.pdf]

SUPPLEMENTAL MATERIAL

Associations of short-term exposure to traffic-related air pollution with hospital admissions in London, U.K.

Evangelia Samoli[1], Richard W Atkisnon[2], Antonis Analitis[1], Gary W Fuller[3], David C Green[3], Ian Mudway [3], H Ross Anderson [2,3], Frank Kelly[3].

1. Department of Hygiene, Epidemiology and Medical Statistics, Medical School, University of Athens, 75 Mikras Asias Str, 115 27 Athens, Greece. 2. MRC-PHE Centre for Environment and Health, St George's, University of London, Cranmer Terrace, London, SW17 0RE U.K. 3. King's College Analytical & Environmental Sciences Division, King's College London, 4th Floor, Franklin-Wilkins Building, 150 Stamford Street, London SE1 9NH U.K.

*Address correspondence to: Evangelia Samoli, Department of Hygiene and Epidemiology, University of Athens Medical School, 75 Mikras Asias Street, 115 27 Athens, Greece Tel:++30-210-7462085, Fax:++30-210-7462205, e-mail: **esamoli@med.uoa.gr**

Annex 1. Selection of traffic-related pollution metrics

The use of indicator species to identify emissions from an air pollution source is well established in receptor analysis and source apportionment. Many studies have used enrichment factors of specific indicator species to identify and quantify sources[1] and indicator species are also used in source apportionment to attribute factors to source types. Viana et al.[2] reviewed PM source apportionment studies conducted across Europe and identified the pollutant species most frequently used in source attribution. These have been used to select indicators of different traffic pollution source mechanisms, along with source specific studies as summarized in Table 1.

Despite being strongly emitted from traffic sources an indicator species might also have other sources in the urban environment. The specificity of the each indicator species will therefore be different. The different transport sources, processes and indicator species are not represented individually in the London Atmospheric Emissions Inventory. We have therefore calculated a kerbside enrichment factor as an index of specificity for London for each tracer during the study period. This has been defined as:

Kerbside enrichment factor = ([Marylebone Road] – [North Kensington]) / [North Kensington]

Enrichment factors have been calculated separately for the warm and cool season to reflect potential differing seasonality between the traffic and non-traffic source types.

Table 1. Source indicators and their kerbside enrichment factors.

**Source**	**Indicator**	**Background**	**Kerbside enrichment factor**
**Traffic - general**	NOx	NOx, the sum of NO and NO_2, is found in greatest concentrations in London close to busy roads. Real-world measurements of exhaust from 72,000 vehicles show greatest NOx emissions arise from diesel and older (pre-EURO 3) petrol vehicles.[3] The London Atmospheric Emissions Inventory shows road transport to be the largest single NOx source in London at 47% of 2010 emissions followed by space heating (16%).[4]	4.6
**Exhaust from petrol vehicles**	CO	CO is emitted from incomplete fuel combustion. Real-world vehicle emissions measurements in London[5] shows exhaust CO between 1.9% for pre-euro petrol cars to 0.07% for Euro 4. By contrast all diesel vehicle types measured had emissions less than 0.07% and some as low as 0.01%.	1.4
**Diesel exhaust**	Black and elemental carbon	The black carbon measurement is a function of the light absorption of particles; which is strongly related to the carbon content of the aerosol. Elemental carbon defines the carbon concentration in particles that is not chemically bound.[6] Europe-wide these are mainly emitted from transport, especially diesel vehicles.[7] Viana et al.[2] list the use of black carbon as a tracer for vehicle exhaust in source apportionment studies. Measurements of	5.6 (for BC)

		real-world vehicle emissions in London using "smoke number" show that diesel vehicles are overwhelmingly the largest emitters with mean smoke numbers from light duty diesels being around 3.5 times greater than those from petrol.[5]	
**Brake wear particles**	Cu	Viana et al.[2] list the use of Cu as an indicator of traffic emissions from brake wear alongside Ba and Sb. The chemical composition of brake linings and brake dust vary according to product and application but due to its use as a high temperature lubricant Cu is generally the most abundant element in brake linings and is found in high abundance in brake dust.[8]	4.7
**Tyre wear particles**	Zn	Tyres are around 1% Zn by weight. It is used as an activator in the vulcanization process and is the only element in tyres that are present at significantly greater than crustal abundance.[8] Viana et al.[2] list the use of Zn as indicator of traffic emissions from tyre wear. Zn has also been used as an indicator of emissions from lubricating oil however traffic emissions are dominated (>90%) by tyre wear sources.[9] This was supported by Harrison et al.[9] who found negligible concentrations of sub-micron Zn in the roadside increment in London.	1.3
**Mineral dust**	Al	Viana et al.[2] list the use of Al, Si, Ca and Fe in PM_{10} as indicators of crustal / mineral particles. Frank[10] used an equation using Si, Ca, Fe and Ti to apportion crustal material. However in urban settings Fe might also originate from vehicle wear sources for instance Harrison et al.[9] used Fe measured in the PM coarse as a marker for vehicle and soil dust in London. Of the remaining metallic elements measured in this study, Al and Ca occur in sufficient quantities to be used as tracers for mineral dusts. Given the identification of Ca in lubricating oil emissions from traffic in London,[11] Al was selected as the favored indicator species.	1.3

References

1. Watson JG, Chow JC, Lowenthal DH, Pritchett LC, Frazier CA, Neuroth GR et al. Differences in the carbon composition of source profiles for diesel-and gasoline-powered vehicles. *Atmos Environ.* 1994; 28:2493-2505.

2. Viana M, Kuhlbusch TAJ, Querol X, Alastuey A, Harrison RM, Hopke PK et al. Source apportionment of particulate matter in Europe: a review of methods and results. *J Aer Scien*. 2008; 39: 827-849.

3. Carslaw DC, Rhys-Tyler G. New insights from comprehensive on-road measurements of NO_X, NO_2 and NH_3 from vehicle emission remote sensing in London, UK. *Atmos Environ* 2013; 81:339-347.

4. Greater London Authority (GLA). 2012. The London Atmospheric Emissions Inventory available at: http://datalondongovuk/dataset/london-atmospheric-emissions-inventory-2010 accessed 19th December 2012.

5. Rhys-Tyler GA, Legassick W, Bell MC. The significance of vehicle emissions standards for levels of exhaust pollution from light vehicles in an urban area. *Atmos Environ.* 2011;45:3286-3293.

6 . Petzold A, Ogren JA, Fiebig M, Laj P,Li SM, Baltensperger U et al. Recommendations for reporting "black carbon" measurements. *Atmos Chem Phys.* 2013; 13: 8365–8379.

7. European Environment Agency (EEA). *Status of black carbon monitoring in ambient air in Europe. EEA technical report 18/2013*. Luxemburg: European Environment Agency; 2013.

8. Thorpe A, Harrison RM. 2008. Sources and properties of non-exhaust particulate matter from road traffic: a review. *STOTEN* 2008;400:270-282.

9. Harrison RM, Jones AM, Gietl J, Yin J, Green DC. Estimation of the contributions of brake dust, tire wear, and resuspension to nonexhaust traffic particles derived from atmospheric measurements. *Environ Sci Tech.* 2012;46:6523-6529.

10. Frank NH. Retained nitrate, hydrated sulfates, and carbonaceous mass in federal reference method fine particulate matter for six eastern US cities. *J Air Waste Manag Ass*, 2006;56: 500-511.

11. Dall'Osto M, Harrison RM. Urban organic aerosols measured by single particle mass spectrometry in the megacity of London. *Atmos Chem Phys.* ,2012;12:4127-4142.

Annex 2. Urban increment estimation

Source apportionment of several metrics was undertaken using the approach first proposed by Lenschow et al.[1] This approach assumes that the measured concentrations in urban areas consist of the sum of contributions from three different source areas, namely:

The regional background: This is the concentration of the pollutant present in air around the city. It is assumed to vary in time, but not space over the city and its surroundings. In the TRAFFIC study measurement sites to the west (Harwell, Oxfordshire), east (Detling, Kent) and, for CO only Egham in Surrey, were used to determine regional concentrations outside the London plume.

The urban background: This concentration is from the sum of all urban sources. The urban increment (urban – regional) is the concentration from urban sources. The urban increment for NOx, CO and BC were included in the analysis as specific tracers for urban traffic sources, as distinct from contributions from industry and more distant sources that determine the regional background. In this study the North Kensington measurement site was used.

The kerb / roadside: This is the concentration from a road source when measured very nearby. The contribution from the nearby road can be deduced from the difference between traffic and urban background monitoring sites. In this study the Marylebone Road measurement site was used to calculate kerbside enrichment factors.

References

1. Lenschow P, Abraham HJ, Kutzner K, Lutz M, Preuß JD, Reichenbächer W. Some ideas about the sources of PM10 *Atmos Environ.* 2001;35: S23-S33.

Annex 3. Supplemental description of the selected pollutants

Table 1. Distribution of the pollutants stratified by warm (April to September) and cool (October to March) period of the year.

	April to September				October to March			
**Pollutant (μg/m^3, except CO mg/m^3)**	**Mean**	**Median**	**IQR[a]**	**90th percentile**	**Mean**	**Median**	**IQR[a]**	**90th percentile**
**NOx**	37.4	31.1	20	63.1	72.9	59.7	55.0	139.0
**NOx - Urban increment**	28.0	21.8	17.1	49.6	56.6	43.9	41.2	136.3
**CO**	0.3	0.3	0.1	0.4	0.4	0.4	0.2	0.6
**CO –Urban Increment**	0.1	0.1	0.1	0.1	0.1	0.1	0.1	0.2
**EC**	0.8	0.6	0.6	1.4	1.3	1.1	0.9	2.4
**EC - Urban**	0.6	0.5	0.3	1.1	0.9	0.7	0.6	1.8
**BC**	1.2	1.0	0.7	2.0	1.8	1.4	1.3	3.4
**BC –Urban**	0.8	0.6	0.4	1.4	1.1	0.9	0.9	2.1
**Cu**	0.0074	0.0063	0.01	0.142	0.0114	0.0087	0.01	0.0228
**Zn**	0.0093	0.0071	0.01	0.060	0.0150	0.0110	0.01	0.0318
**Al**	0.0765	0.0552	0.07	0.1555	0.0751	0.0567	0.06	0.1458
**PM_{10}**	15.2	13.1	8.0	24.0	21.5	18.0	12.5	38.0
**$PM_{2.5}$**	8.9	7.3	4.1	14.8	15.5	11.0	12.1	32.0
**NO_2**	28.9	26.1	14.5	45.2	43.5	43.0	22.9	62.9
**SO_2**	1.60	1.7	2.4	3.0	2.1	2.0	2.1	4.1
**O_3**	70.1	67.2	26.9	97.6	41.0	44.0	26.3	63.7

[a]IQR: Interquartile Range

Table 2. Annual correlations between pollutants.

Pollutant	NOx	NOx-Urban	CO	CO-Urban	EC	EC-Urban	BC	BC-Urban	C	Zn	Al	PM_{10}	$PM_{2.5}$	NO_2	SO_2
**NOx**	1														
**NOx – Urban**	0.98	1													
**CO**	0.83	0.81	1												
**CO –Urban**	0.35	0.41	0.60	1											
**EC**	0.91	0.90	0.74	0.37	1										
**EC - Urban**	0.78	0.83	0.62	0.45	0.92	1									
**BC**	0.90	0.88	0.77	0.36	0.92	0.78	1								
**BC –Urban**	0.81	0.83	0.66	0.35	0.86	0.85	0.92	1							
**Cu**	0.77	0.76	0.62	0.32	0.81	0.69	0.78	0.65	1						
**Zn**	0.68	0.63	0.57	0.13	0.68	0.45	0.68	0.45	0.74	1					
**Al**	0.36	0.33	0.26	0.00	0.40	0.28	0.40	0.26	0.49	0.55	1				
**PM_{10}**	0.65	0.57	0.57	0.04	0.53	0.32	0.60	0.38	0.56	0.71	0.65	1			
**$PM_{2.5}$**	0.65	0.57	0.58	0.05	0.54	0.33	0.61	0.39	0.56	0.71	0.55	0.95	1		
**NO_2**	0.90	0.87	0.74	0.23	0.83	0.69	0.80	0.72	0.71	0.66	0.44	0.66	0.66	1	
**SO_2**	0.55	0.51	0.42	0.08	0.46	0.24	0.48	0.32	0.40	0.47	0.37	0.51	0.49	0.52	1
**O_3**	-0.48	-0.45	-0.42	-0.13	-0.36	-0.23*	-0.39	-0.39	-0.27	-0.23	0.19	-0.19	-0.28	-0.40	-0.17

Annex 4. Percent change (and 95% confidence intervals (CIs)) in cardiovascular and respiratory hospital admissions associated with an interquartile range increase (in μg/m^3) in regulated pollutants following single day (lag 1 for cardiovascular and lag 2 for respiratory diagnoses) or weekly exposure (lags 0-6) in London, U.K. for 2011–12.

**Pollutants**	**CVD Admissions % (95% CI)**		**Respiratory Admissions % (95% CI)**		
	**15-64 years**	**65+ years**	**0-14 years**	**15-64 years**	**65+ years**
**Single Day Exposure**					
**PM_{10}**	0.17 (-0.86, 1.21)	-0.50 (-1.27, 0.28)	0.69 (-0.85, 2.25)	-0.67 (-1.69, 0.37)	-1.14 (-2.10, -0.16)
**$PM_{2.5}$**	0.19 (-0.73, 1.12)	-0.80 (-1.49, -0.10)	0.42 (-0.94, 1.81)	-0.80 (-1.72, 0.13)	-0.97 (-1.84, -0.09)
**NO_2**	1.00 (-0.87, 2.91)	-0.75 (-2.15, 0.68)	1.91 (-0.78, 4.67)	-1.14 (-2.94, 0.69)	-3.11 (-4.75, -1.44)
**SO_2**	0.79 (-0.72, 2.32)	-0.15 (-1.29, 1.00)	0.69 (-1.38, 2.81)	-0.90 (-2.38, 0.61)	-1.93 (-3.26, -0.57)
**O_3**	-0.12 (-2.01, 1.81)	1.58 (0.13, 3.05)	-1.64 (-4.35, 1.15)	3.23 (1.31, 5.19)	4.83 (3.01, 6.68)
**Weekly Exposure**					
**PM_{10}**	-0.76 (-2.32, 0.81)	-0.32 (-1.50, 0.87)	2.59 (-0.24, 5.50)	-0.61 (-2.17, 0.98)	-3.28 (-4.80, -1.73)
**$PM_{2.5}$**	-0.63 (-2.07, 0.83)	-0.66 (-1.74, 0.43)	2.21 (-0.33, 4.81)	-0.92 (-2.36, 0.54)	-3.03 (-4.43, -1.60)
**NO_2**	-1.52 (-4.47, 1.53)	-0.16 (-2.42, 2.16)	6.30 (1.07, 11.81)	-1.69 (-4.65, 1.35)	-7.16 (-9.89, -4.35)
**SO_2**	-1.32 (-4.07, 1.51)	-0.35 (-2.44, 1.79)	4.95 (0.54, 9.56)	-1.99 (-4.75, 0.87)	-3.21 (-5.77, -0.58)
**O_3**	0.66 (-2.22, 3.63)	1.95 (-0.23, 4.19)	-7.89 (-12.39, -3.16)	4.71 (1.66, 7.85)	8.84 (5.73, 12.04)

Annex 5. Results from multi pollutants' models. Percent change (and 95% confidence intervals (CIs)) in cardiovascular and respiratory hospital admissions associated with an interquartile range increase in traffic pollutants after single day exposure (lag 1 for cardiovascular and lag 2 for respiratory diagnoses) in London, U.K. for 2011–12. EC/BC and metals are also adjusted for PM mass.

**Indicator/ Pollutant**	**Controlling for**	**Cardiovascular Admissions % (95%CI)**		**Respiratory Admissions % (95%CI)**		
		**15-64 years**	**65+ years**	**0-14 years**	**15-64 years**	**65+ years**
**General traffic**						
NOx	O_3	1.05 (-0.27, 2.38)	0.24 (-0.76, 1.25)	0.74 (-1.01, 2.52)	0.12 (-1.14, 1.40)	-0.59 (-1.74, 0.57)
	SO_2	0.74 (-0.65, 2.16)	-0.38 (-1.44, 0.69)	0.96 (-0.85, 2.80)	-0.62 (-1.99, 0.77)	-1.42 (-2.66, -0.16)
	$PM_{2.5}$	1.22 (-0.26, 2.73)	0.54 (-0.59, 1.69)	0.98 (-0.88, 2.88)	-0.31 (-1.73, 1.13)	-1.65 (-2.92, -0.36)
NOx Urban	O_3	1.07 (-0.13, 2.29)	0.36 (-0.56, 1.28)	0.82 (-0.75, 2.41)	0.22 (-0.93, 1.39)	-0.47 (-1.53, 0.59)
	SO_2	0.77 (-0.48, 2.05)	-0.14 (-1.10, 0.83)	0.94 (-0.68, 2.58)	-0.42 (-1.65, 0.84)	-1.12 (-2.25, 0.02)
	$PM_{2.5}$	1.12 (-0.17, 2.41)	0.53 (-0.45, 1.52)	0.97 (-0.64, 2.61)	-0.17 (-1.40, 1.08)	-1.27 (-2.38, -0.14)
	EC Urban	-2.02 (-4.79, 0.84)	0.91 (-1.33, 3.20)	-0.24 (-4.03, 3.70)	-2.78 (-5.58, 0.10)	-2.12 (-4.68, 0.52)
**Petrol vehicle exhaust**						
CO	O_3	1.86 (0.29, 3.46)	-0.22 (-1.42, 0.98)	0.79 (-1.39, 3.01)	-0.37 (-1.93, 1.22)	-0.85 (-2.28, 0.61)
	SO_2	1.70 (0.05, 3.39)	-0.62 (-1.88, 0.65)	0.70 (-1.54, 3.00)	-1.12 (-2.77, 0.56)	-1.69 (-3.20, -0.15)

	$PM_{2.5}$	2.09 (0.31, 3.91)	0.19 (-1.17, 1.56)	0.95 (-1.40, 3.35)	-0.61 (-2.37, 1.17)	-1.80 (-3.38, -0.20)
CO Urban	*O_3*	0.96 (-0.07, 2.00)	-0.07 (-0.85, 0.72)	0.94 (-0.44, 2.35)	0.37 (-0.67, 1.41)	-0.32 (-1.25, 0.62)
	SO_2	1.02 (-0.03, 2.07)	-0.20 (-1.00, 0.60)	0.81 (-0.60, 2.24)	0.17 (-0.88, 1.23)	-0.48 (-1.43, 0.49)
	$PM_{2.5}$	0.96 (-0.07, 1.99)	-0.06 (-0.84, 0.72)	0.91 (-0.47, 2.31)	0.29 (-0.74, 1.32)	-0.49 (-1.42, 0.46)
	EC Urban	0.43 (-0.87, 1.75)	0.02 (-0.99, 1.03)	0.38 (-1.38, 2.17)	0.26 (-1.04, 1.59)	-0.60 (-1.77, 0.58)
**Diesel vehicle exhaust**						
EC	*NOx*	4.15 (0.88, 7.53)	0.54 (-1.91, 3.05)	-0.42 (-4.87, 4.24)	0.13 (-3.03, 3.39)	1.16 (-1.85, 4.27)
	CO	0.90 (-1.05, 2.90)	0.38 (-1.11, 1.90)	0.79 (-1.82, 3.47)	0.32 (-1.62, 2.30)	-0.17 (-1.96, 1.65)
EC Urban	*NOx*	2.49 (0.57, 4.45)	0.05 (-1.42, 1.55)	1.65 (-1.08, 4.45)	2.09 (0.17, 4.06)	1.63 (-0.15, 3.45)
	CO	1.00 (-0.37, 2.38)	0.21 (-0.84, 1.28)	0.97 (-0.87, 2.85)	0.73 (-0.63, 2.12)	0.72 (-0.53, 1.99)
BC	*NOx*	3.32 (-0.22, 6.98)	0.33 (-2.31, 3.03)	-1.32 (-6.18, 3.79)	-0.60 (-4.01, 2.92)	2.90 (-0.45, 6.37)
	CO	0.40 (-1.74, 2.59)	0.74 (-0.91, 2.41)	0.68 (-2.14, 3.59)	0.65 (-1.51, 2.86)	-0.01 (-2.02, 2.03)
BC Urban	*NOx*	0.55 (-1.66, 2.82)	-0.63 (-2.31, 1.08)	0.59 (-2.48, 3.76)	0.28 (-1.91, 2.52)	1.85 (-0.19, 3.94)
	CO	-0.21 (-1.74, 1.35)	0.00 (-1.18, 1.19)	0.64 (-1.40, 2.71)	0.76 (-0.78, 2.32)	0.79 (-0.60, 2.21)
**Vehicle Non-exhaust**						
Cu	*NOx*	1.37 (-0.56, 3.34)	0.03 (-1.45, 1.53)	-1.40 (-3.97, 1.25)	-1.55 (-3.45, 0.38)	-1.07 (-2.84, 0.74)
	CO	0.80 (-0.85, 2.47)	0.15 (-1.11, 1.42)	-0.27 (-2.47, 1.99)	-1.16 (-2.80, 0.51)	-1.31 (-2.82, 0.23)

ZN	*NOx*	-0.27 (-1.73, 1.22)	0.17 (-0.96, 1.31)	-1.85 (-3.85, 0.20)	-0.44 (-1.94, 1.09)	-0.14 (-1.51, 1.24)
	CO	-0.38 (-1.76, 1.03)	0.21 (-0.85, 1.28)	-1.16 (-3.05, 0.77)	-0.18 (-1.6, 1.26)	-0.40 (-1.69, 0.91)
Al	*NOx*	0.55 (-1.08, 2.21)	-1.13 (-2.37, 0.12)	-0.39 (-2.84, 2.11)	0.79 (-0.89, 2.50)	1.62 (0.06, 3.21)
	CO	0.39 (-1.22, 2.03)	-1.13 (-2.35, 0.10)	0.20 (-2.22, 2.68)	0.86 (-0.79, 2.55)	1.45 (-0.09, 3.01)

Annex 6. Percent change (and 95% confidence intervals (CIs)) in hospital admissions by age group and season associated with season-specific interquartile range (IQR) increase in traffic-related pollutants after single day exposure (lag 1 for cardiovascular (A) and lag 2 for respiratory (B) diagnoses) in London, U.K. for 2011–12. EC/BC and metals are adjusted for PM mass.

(A) Cardiovascular Admissions

**Indicator/Pollutants**	**15-64 years % (95% CI)**		**65+ years % (95% CI)**	
	**April-September**	**October-March**	**April-September**	**October-March**
**General traffic**				
NOx	0.87 (-0.96, 2.73)	0.53 (-1.09, 2.17)	-0.59 (-1.94, 0.78)	0.07 (-1.24, 1.40)
NOx – Urban	1.12 (-0.62, 2.89)	0.41 (-0.99, 1.84)	-0.40 (-1.69, 0.90)	0.13 (-1.01, 1.29)
**Petrol vehicle exhaust**				
CO	0.95 (-1.08, 3.02)	0.96 (-0.67, 2.61)	-0.63 (-2.14, 0.90)	-0.07 (-1.39, 1.27)
CO –Urban	0.82 (-0.69, 2.36)	0.31 (-1.20, 1.84)	-0.22 (-1.34, 0.92)	0.02 (-1.21, 1.26)
**Diesel vehicle exhaust**				
EC	2.41 (-0.03, 4.90)	0.56 (-1.30, 2.46)	-0.66 (-2.47, 1.18)	0.00 (-1.51, 1.54)
EC - Urban	1.23 (-0.16, 2.63)	0.33 (-1.25, 1.94)	-0.28 (-1.33, 0.78)	-0.21 (-1.53, 1.13)
BC	3.22 (0.75, 5.74)	0.85 (-1.37, 3.11)	-0.10 (-1.91, 1.74)	0.66 (-1.14, 2.49)
BC –Urban	1.92 (0.08, 3.80)	0.68 (-1.32, 2.73)	0.04 (-1.34, 1.44)	-0.19 (-1.78, 1.43)

Vehicle Non-exhaust

Cu	1.33 (-0.99, 3.70)	1.51 (-0.66, 3.73)	-0.28 (-1.97, 1.45)	-0.32 (-2.13, 1.51)
Zn	0.77 (-1.30, 2.87)	0.07 (-2.17, 2.37)	0.06 (-1.44, 1.58)	0.14 (-1.76, 2.07)
Al	-0.06 (-3.13, 3.11)	0.23 (-1.85, 2.36)	-0.37 (-2.65, 1.97)	-2.12 (-3.84, -0.38)

(B) Respiratory Admissions

	0-14 years % (95%CI)		15-64 years % (95%CI)		65+ years % (95%CI)	
	**April-September**	**October-March**	**April-September**	**October-March**	**April-September**	**October-March**
**General traffic**						
NOx	1.14 (-2.26, 4.67)	2.40 (0.09, 4.76)	0.47 (-1.40, 2.38)	-0.65 (-2.30, 1.02)	0.57 (-0.93, 2.08)	-2.09 (-3.75, -0.39)
NOx – Urban	1.41 (-1.89, 4.81)	2.14 (0.14, 4.17)	0.40 (-1.41, 2.23)	-0.32 (-1.75, 1.13)	0.46 (-0.98, 1.92)	-1.60 (-3.06, -0.12)
**Petrol vehicle exhaust**						
CO	0.52 (-3.18, 4.36)	1.62 (-0.82, 4.12)	0.33 (-1.71, 2.42)	-0.20 (-1.93, 1.55)	1.19 (-0.44, 2.85)	-1.13 (-2.89, 0.67)
CO –Urban	0.24 (-2.49, 3.05)	0.84 (-1.43, 3.16)	-0.37 (-1.87, 1.16)	1.41 (-0.19, 3.03)	-0.31 (-1.52, 0.90)	0.33 (-1.32, 2.01)
**Diesel vehicle exhaust**						
EC	5.67 (0.92, 10.64)	0.93 (-1.80, 3.73)	0.58 (-1.94, 3.16)	0.39 (-1.50, 2.31)	-0.57 (-2.58, 1.49)	-0.93 (-2.87, 1.05)
EC - Urban	3.81 (1.02, 6.68)	2.10 (-0.19, 4.44)	0.52 (-0.98, 2.05)	-0.31 (-1.81, 1.22)	0.05 (-1.16, 1.27)	-0.76 (-2.42, 0.92)
BC	2.48 (-2.15, 7.33)	1.86 (-1.23, 5.04)	0.41 (-2.09, 2.97)	0.66 (-1.62, 3.00)	-0.51 (-2.48, 1.50)	-1.16 (-3.48, 1.21)
BC –Urban	1.04 (-2.42, 4.63)	2.89 (0.04, 5.83)	-0.18 (-2.06, 1.75)	0.99 (-1.05, 3.07)	-0.47 (-1.91, 0.98)	-0.27 (-2.35, 1.86)

**Vehicle Non-exhaust**						
Cu	1.54 (-2.92, 6.22)	-1.53 (-4.65, 1.70)	-1.31 (-3.72, 1.17)	-0.76 (-3.13, 1.66)	-1.01 (-2.91, 0.92)	-2.26 (-4.64, 0.18)
Zn	1.55 (-2.21, 5.45)	-3.99 (-7.30, -0.56)	0.48 (-1.56, 2.57)	-0.64 (-3.18, 1.97)	-0.21 (-1.80, 1.41)	-1.48 (-4.05, 1.16)
Al	4.39 (-1.17, 10.26)	-2.06 (-5.46, 1.45)	-0.14 (-3.12, 2.94)	-0.90 (-3.34, 1.60)	1.25 (-1.08, 3.63)	-0.30 (-2.80, 2.27)
